# *Enterocytozoon bieneusi* in patients with diarrhea and in animals in the northeastern Chinese city of Yichun: genotyping and assessment of potential zoonotic transmission[Fn FN1]

**DOI:** 10.1051/parasite/2022041

**Published:** 2022-09-01

**Authors:** Kexin Zhou, Mingchao Liu, Yanchen Wu, Ran Zhang, Ru Wang, Hui Xu, Yujia Wang, Lan Yao, Hongmei Yu, Aiqin Liu

**Affiliations:** 1 Department of Parasitology, Harbin Medical University Harbin 150081 Heilongjiang China; 2 Central Hospital of Yichun Forestry Administration Yichun 153000 Heilongjiang China

**Keywords:** *Enterocytozoon bieneusi*, ITS region, Genotyping, Diarrheal patients, Animals

## Abstract

*Enterocytozoon bieneusi* is a common microsporidia species in humans and animals. Due to lack of effective vaccines and drugs, understanding of its epidemiological status and characteristics in different hosts is an important step in controlling the infection. The present study aimed at determining the prevalence of *E. bieneusi* in humans with diarrhea and animals in Yichun, in northeastern China, and assessing the epidemiological role of animals in the transmission of microsporidiosis. A total of 540 fecal samples were collected from diarrheal patients (*n* = 222) and 11 animal species (*n* = 318). *Enterocytozoon bieneusi* was identified and genotyped by polymerase chain reaction (PCR) and sequencing of the internal transcribed spacer (ITS) region of the rRNA gene. *Enterocytozoon bieneusi* was detected in 1.4% (3/222) of diarrheal patients, and genotype D and novel genotypes YCHH1 and YCHH2 were identified. *Enterocytozoon bieneusi* was detected in wild boars (7.7%), sika deer (8.2%), dogs (3.2%), and ostriches (10.7%), and genotypes D, Type IV, Peru6, BEB6 and novel genotypes YCHA1, YCHA2 and YCHA3 were identified. Genotypes YCHH1, YCHH2 and YCHA1 were phylogenetically assigned to group 1, while YCHA2 and YCHA3 to groups 2 and 11, respectively. The finding of genotype D in humans and animals, and the identification of zoonotic genotypes Peru6, Type IV, BEB6 in animal-derived *E. bieneusi* isolates indicate the potential of zoonotic transmission of microsporidiosis in the investigated area. The observation of the three novel genotypes in group 1 indicates their zoonotic potential.

## Abstract (fr)

*Enterocytozoon bieneusi* est une espèce de microsporidie commune chez les humains et les animaux. En raison du manque de vaccins et de médicaments efficaces, la compréhension de son statut épidémiologique et de ses caractéristiques chez différents hôtes est une étape importante dans le contrôle de l’infection. La présente étude visait à déterminer la prévalence d’*E. bieneusi* chez les humains souffrant de diarrhée et les animaux à Yichun, dans le nord-est de la Chine, et à évaluer le rôle épidémiologique des animaux dans la transmission de la microsporidiose. Cinq cent quarante échantillons fécaux ont été prélevés chez des patients diarrhéiques (*n* = 222) et 11 espèces animales (*n* = 318). *Enterocytozoon bieneusi* a été identifié et génotypé par PCR et séquençage de la région de l’espaceur interne transcrit (ITS) du gène de l’ARNr. *Enterocytozoon bieneusi* a été détecté chez 1,4 % (3/222) des patients souffrant de diarrhée, et le génotype D et les nouveaux génotypes YCHH1 et YCHH2 ont été identifiés. *Enterocytozoon bieneusi* a été détecté chez des sangliers (7,7 %), des cerfs sika (8,2 %), des chiens (3,2 %) et des autruches (10,7 %), et les génotypes D, Type IV, Peru6, BEB6 et les nouveaux génotypes YCHA1, YCHA2 et YCHA3 ont été identifiés. Les génotypes YCHH1, YCHH2 et YCHA1 ont été phylogénétiquement assignés au groupe 1, et YCHA2 et YCHA3 respectivement aux groupes 2 et 11. La découverte du génotype D chez les humains et les animaux et l’identification des génotypes zoonotiques Peru6, Type IV, BEB6 dans les isolats d’*E. bieneusi* d’origine animale indiquent le potentiel de transmission zoonotique de la microsporidiose dans la zone étudiée. L’observation des trois nouveaux génotypes dans le groupe 1 indique leur potentiel zoonotique.

## Introduction

*Enterocytozoon bieneusi* is a common intestinal pathogen in humans worldwide and has been reported in humans in more than 20 countries [60]. It has also been found in approximately 170 animal species (mammals, birds, reptiles and insects) in more than 40 countries, displaying a wide geographical distribution and a zoonotic nature [60]. *Enterocytozoon bieneusi* causes a disease characterized mainly by diarrhea. Diarrhea is usually self-limiting in immunocompetent individuals, but life-threatening in immunocompromised/immunodeficient individuals, such as HIV patients [29]. Infections commonly appear asymptomatic in animal hosts [39].

Most *E. bieneusi* infections are via fecal-oral transmission of infective spores from infected humans and animals through contaminated water or food [7]. This pathogen has been detected in multiple water supplies as a potential transmission vehicle, such as irrigation waters, recreational waters, and treated raw- and wastewaters [21]. A waterborne outbreak of intestinal microsporidiosis related to *E. bieneusi* occurred in France in 1995 [4]. Studies have also identified *E. bieneusi* in vegetables, fruits and milk [15, 17]. Two foodborne outbreaks of microsporidiosis due to *E. bieneusi* were reported in Sweden in 2009 and Denmark in 2020 [6, 30].

*Enterocytozoon bieneusi* has been confirmed to be a genetically complex species. To date, based on sequence analysis of the internal transcribed spacer (ITS) region with a high degree of genetic diversity in the rRNA gene, ~90 and ~600 genotypes of *E. bieneusi* have been identified in humans and animals, respectively [60]. These genotypes can be further phylogenetically divided into 11 different groups and an outlier [21]. Group 1 is the largest and the most complicated, which is composed of almost all the genotypes from humans and the majority of genotypes from animal hosts [21]. The fact that some genotypes co-occur in humans and animals supports the potential for zoonotic or cross-species transmission, especially for genotypes D, EbpC, and Type IV which exhibit remarkable adaption to life within a diverse array of host and natural environments [23]. Group 2 is the second largest group, which was previously considered to be adapted to ruminants [22]. However, some genotypes in group 2 (notably BEB4, BEB6, CHN3, I and J) have been found in multiple animal species and humans, raising public health concern related to the zoonotic potential of this group [22]. Current available data imply host adaptation in most genotypes in groups 3–11 as well as the three outlier genotypes, showing their limited or minimal effects on public health [21].

In China, *E. bieneusi* was first identified in both humans and animals in 2011 [55]. To date, epidemiological studies of *E. bieneusi* have been carried out in humans in 10 provinces, municipalities and autonomous regions. More than 7000 people have been involved in investigational studies of *E. bieneusi* [12, 18, 36, 52, 53]*.* Prevalences varied by different populations: 0.2%–22.5% for diarrheal children; 4.2%–7.5% for children without gastrointestinal diseases; 1.2% for children with different disease backgrounds; 5.0%–13.2% for diarrheal adults; 6.5%–8.1% for non-diarrheal adults; 5.7%–11.6% for HIV-positive patients; 4.3% for HIV-negative patients; 1.3% for cancer patients [8, 18, 24, 25, 36, 45, 46, 48, 51–55]. A total of 66 ITS genotypes were identified out of 280 *E. bieneusi*-positive specimens: 54 in group 1, nine in group 2, and three in group 5. Among them, 30 genotypes from 86.1% (241/280) of positive specimens have also been found in animals, indicating potential zoonotic transmission (Supplementary Table S1). The parasite has been detected in various animals distributed in 24 provinces/autonomous regions/municipalities of China, and zoonotic genotypes are frequently identified among animal-derived *E. bieneusi* isolates [37]. In an epidemiological investigation of *E. bieneusi* in cattle conducted in Jilin Province, the percentage of zoonotic genotypes was reported to be high, up to 100% [55]. In northeastern China’s Heilongjiang Province, although epidemiological studies of *E. bieneusi* have been reported in humans and animals, no data are available about *E. bieneusi* infection in Yichun, in the northeasternmost part of the province. The present study determined the prevalence of *E. bieneusi* infection in humans with diarrhea and in animals, and assessed the epidemiological role of animals in the transmission of microsporidiosis caused by *E. bieneusi* by polymerase chain reaction (PCR) amplification and sequence analysis of the ITS region of the rRNA gene.

## Material and methods

### Ethics statement

The present study protocol was reviewed and approved by the Ethics Committees of Harbin Medical University and Central Hospital of Yichun Forestry Administration. We explained our study objectives and procedures to all the adult participants and the parents/guardians when the participants under the age of 18. The same explanation was given to the managers or/and the owners of animals before beginning our study to have their animals involved in the present study. All the animal fecal samples were collected only after defecation without disturbing them.

### Study sites and collection of fecal samples

A molecular epidemiological investigation of *E. bieneusi* was carried out in humans and animals in Yichun City (46°28′–49°26′ N, 127°37′–130°46′ E), Heilongjiang Province, which faces Russia across the Wusuli River. During the period from October 2018 to June 2021, a total of 540 fecal samples (222 from humans and 318 from animals) were collected. Human fecal samples were from diarrheal patients (one from each) in the Central Hospital of Yichun Forestry Administration, comprising children (aged < 5, *n* = 26), minors (aged 5–17, *n* = 34), young adults (aged 18–35, *n* = 31), middle-aged adults (aged 36–60, *n* = 83) and older adults (aged > 60, *n* = 48). The immune status of the patients was not known. Animal fecal samples were from mammals (*n* = 225) and birds (*n* = 93). Wild boars (*n* = 13), ostriches (*n* = 28), red shelducks (*n* = 5), pigeons (*n* = 5), turkeys (*n* = 14), bar-headed geese (*n* = 5), peacocks (*n* = 21) and emus (*n* = 15) were from a scenic area – Jiufeng Mountain Yangxin Valley, in which at least 1400 mammals and 500 birds are fed for tourists to watch. Sika deer (*n* = 110) were from Jinshan Deer Park. The park is a natural deer farm in a valley, in which approximate 500 deer are raised under semi free-grazing conditions. No animals mentioned above had gastrointestinal symptoms at the time of the sampling. Dogs (*n* = 62) and cats (*n* = 40) were from Yifeng Pet Hospital, with one dog and two cats suffering from diarrhea at the time of sampling. All the fecal samples were stored in a refrigerator at 4 °C (≤2 days) or −20 °C (>2 days) prior to being used in the subsequent molecular analysis.

### DNA extraction and PCR amplification

Genomic DNA was directly extracted from 180–200 mg fecal samples using a QIAamp DNA Mini Stool Kit (Qiagen, Hilden, Germany), according to manufacturer-recommended procedures. The extracted DNA was eluted in 200 μL of AE and stored at −20 °C in a freezer prior to PCR analysis.

All the DNA preparations were detected for the presence of *E. bieneusi* by nested PCR amplification of approximately 410 bp nucleotide fragment of the rRNA gene, which is composed of 79 bp of the 3′-end of the small subunit rRNA (SSU rRNA) gene, 243 bp of the ITS region and 87 bp of 5′-region of the large subunit rRNA (LSU rRNA) gene. The primer sequences and the cycling parameters in PCR analysis were used as previously described [31]. TaKaRa Taq DNA Polymerase (TaKaRa Bio Inc., Tokyo, Japan) was used for all the PCR amplifications. A negative control with no DNA added and a positive control with DNA of a rabbit-derived genotype CHN-RD1 were included in all PCR tests. All the secondary PCR products were subjected to electrophoresis in a 1.5% agarose gel and visualized by staining the gel with GelStrain (TransGen Biotech., Beijing, China).

### Nucleotide sequencing and analyzing

All the secondary PCR products of the anticipated size were directly sequenced by Comate Bioscience Company Limited (Jilin, China), with the secondary PCR primers using the BigDyeTerminator v3.1Cycle Sequencing Kit (Applied Bio systems, Carlsbad, CA, USA) on an ABI Prism 3730 XL DNA Analyzer. Accuracy of the sequencing results was ensured by bi-directional sequencing. Nucleotide sequences obtained in the present study were subjected to BLAST searches (http://www.ncbi.nlm.nih.gov/blast/), and then aligned and analyzed with each other and reference nucleotide sequences deposited in GenBank database using Clustal X 1.81 (http://www.clustal.org/). If the nucleotide sequences were identical to known genotypes, the first published name would be given according to the established nomenclature system [38]. If the nucleotide sequences were different from published sequences and were confirmed to be novel sequences by sequencing another two separate PCR products of the same preparations, they represented novel genotypes.

### Phylogenetic and statistical analyses

To better present the genetic diversity of all the genotypes of *E. bieneusi* obtained in the present study and to assess the genetic relationship of the novel ones here to the known ones, a phylogenetic analysis was performed. All the aligned ITS sequences of *E. bieneusi* were implemented into Mega 5 software (http://www.megasoftware.net/), and a neighbor-joining tree was constructed based on evolutionary distances calculated by a Kimura 2-parameter model. The reliability of these trees was assessed using bootstrap analysis with 1000 replicates.

To determine the relationships between the prevalences of *E. bieneusi* and the variables, statistical analyses were performed by using Pearson chi-square (*χ*^2^) and Fisher’s exact tests based on the Statistical Package for the Social Sciences (SPSS). Differences were considered statistically significant at *p* < 0.05.

## Results

### Prevalence of *E. bieneusi*

A total of 540 fecal samples were screened for the presence of *E. bieneusi* by PCR amplification and sequence analysis of the ITS region of the rRNA gene. Three human fecal samples were positive for *E. bieneusi* (1.4%, 3/222), distributing in three age groups: <5 years (1/26, 3.9%); 5–17 years (1/34, 2.9%); >60 years (1/48, 2.1%). All three cases of *E. bieneusi* infection were from males without contact with animals. None of the differences in prevalence of *E. bieneusi* were statistically significant by each of the variables – age, gender and contact with animals (*p* > 0.05) (Table 1). Among the animal fecal samples investigated, *E. bieneusi* was only detected in three mammal species – wild boars (1/13, 7.7%), sika deer (9/110, 8.2%) and dogs (2/62, 3.2%), and one bird species –ostriches (3/28, 10.7%) (Table 2).

**Table 1:** Assessment of possible risk factors for human *E*. *bieneusi* infection in Yichun.

Variable	Examined no.^a^	Positive no. (%)	OR (95% CI)	*χ*2/*p*-value
Age (years)				
Children (<5)	26	1 (3.9)	Ref.^b^	
Minors (5–17)	34	1 (2.9)	1.32 (0.08, 22.15)	0.00/1.00
Young adults (18–35)	31	0	0.96 (0.89, 1.04)	–/0.46
Middle aged adults (36–60)	83	0	0.96 (0.89, 1.04)	–/0.24
Older adults (>60)	48	1 (2.1)	1.88 (0.11, 31.35)	0.00/1.00
Gender				
Male	121	3 (2.5)	0.98 (0.95, 1.00)	0.91/0.34
Female	94	0		
Contact with animals				
Yes	30	0	1.02 (1.00, 1.04)	–/1.00
No	185	3 (1.6)		

a Only 215 participants provided information other than age.

b The children age group (<5 years) is used as the reference group for comparison with the prevalences of the other four age groups.

**Table 2:** Prevalence and genotype distribution of *E*. *bieneusi* in humans and animals in Yichun.

Host		No. positive/No. examined (%)	ITS genotype (*n*)		Accession no.^b^
			Known	Novel	
Humans		3/222 (1.4)	D (1)	YCHH1 (1); YCHH2 (1)	OL333420; OL333421
Animals					
Mammals	Wild boars	1/13 (7.7)		YCHA1 (1)	OL333422
	Sika deer	9/110 (8.2)	D (2); BEB6 (6); Peru6 (1)		
	Dogs	2/62 (3.2)		YCHA2 (1); YCHA3 (1)	OL333423; OL333424
	Cats	0/40 (0)			
Birds	Ostriches	3/28 (10.7)	Type IV (1); D (2)		
	Others^a^	0/65 (0)			

a Including red shelducks (*n* = 5), pigeons (*n* = 5), turkeys (*n* = 14), bar-headed geese (*n* = 5), peacocks (*n* = 21) and emus (*n* = 15).

b Only accession nos. of the five novel genotypes obtained in the present study.

### Genetic characterization and host distribution of *E. bieneusi* genotypes

Sequence analysis of the ITS region of the rRNA gene identified nine genotypes out of 18 *E. bieneusi*-positive samples: four known genotypes including D (*n* = 5), Type IV (*n* = 1), Peru6 (*n* = 1) and BEB6 (*n* = 6), and five novel genotypes named as YCHH1, YCHH2, YCHA1, YCHA2, YCHA3 (one each) (GenBank: OL333420–OL333424). The five novel genotypes had 99.6%, 99.6%, 99.2%, 99.6% and 98.8% homology with the genotypes D (AF101200), EbpA (AF076040), D (AF101200), BEB6 (EU153584) and PtEb IX (DQ885585), respectively with one to three base varieties being found at the ITS region of the rRNA gene.

Among the known genotypes, genotype D had a broad host distribution, which could be found in one human, two sika deer and two ostriches. Genotype BEB6 showed predominance in sika deer, accounting for 66.7% (6/9) of all *E. bieneusi* isolates belonging to this genotype. Host distribution of all *E*. *bieneusi* genotypes is shown in Table 2.

### Phylogenetic relationship of *E. bieneusi* genotypes

**Figure 1:**
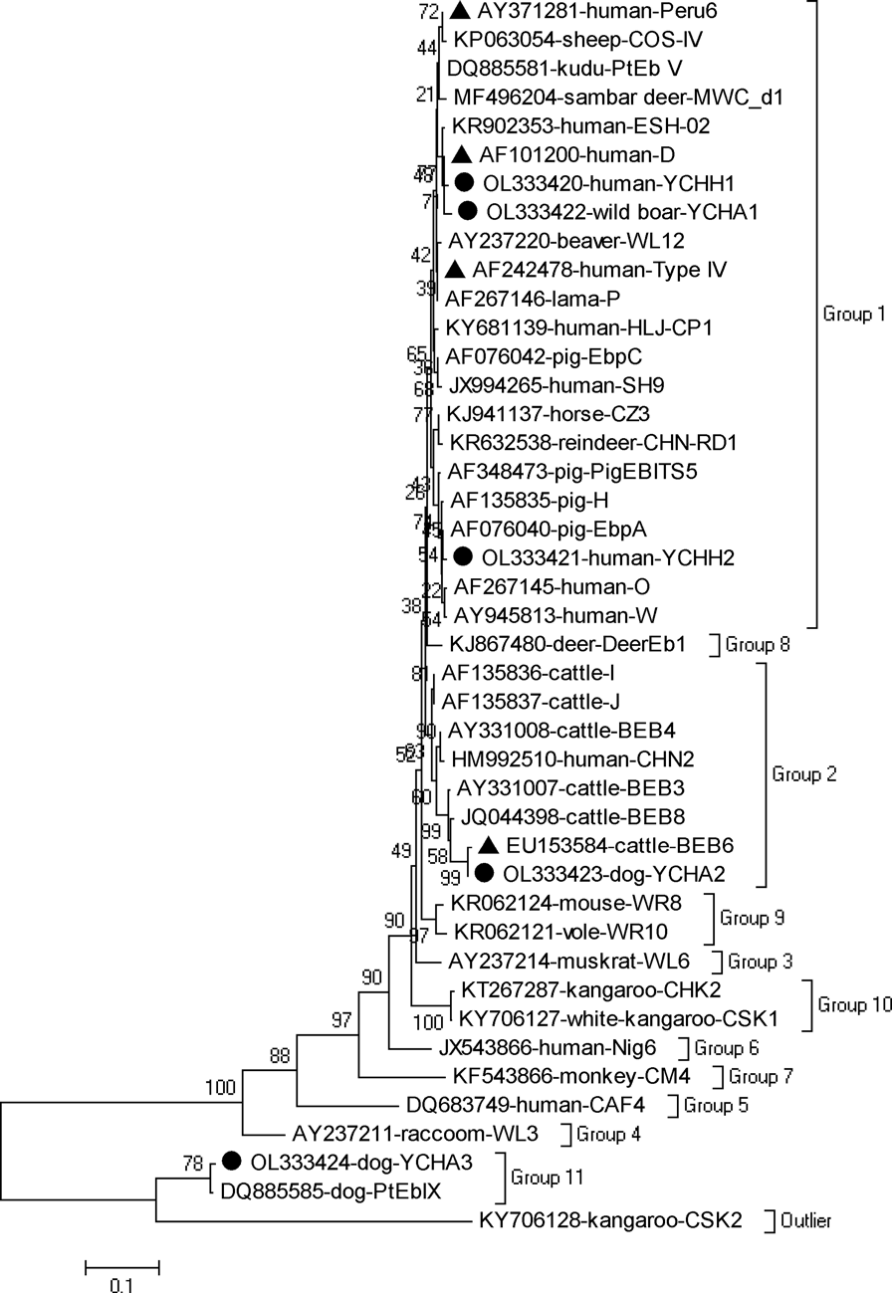
Phylogenetic relationships of the *E. bieneusi* genotypes of *E. bieneusi* identified in this study and other known genotypes as inferred by a neighbor-joining analysis of ITS sequences based on genetic distances calculated by the Kimura 2-parameter model. The numbers on the branches are percent bootstrapping values from 1000 replicates. Each nucleotide sequence is identified by its accession number, host origin, and genotype designation. The black triangles and circles are known and novel genotypes identified in this study, respectively.

In a phylogenetic analysis of the ITS sequences of *E. bieneusi*, the nine genotypes obtained in the present study fell into three different genetic groups: genotypes D, Type IV, Peru6, YCHH1, YCHH2 and YCHA1 in group 1 with a zoonotic nature; genotypes BEB6 and YCHA2 in group 2 with increasing zoonotic potential; and genotype YCHA3 in group 11 mainly specific for dogs (Fig. 1).

## Discussion

*Enterocytozoon bieneusi* is recognized as one of the causative agents responsible for diarrhea; however, it is often underestimated or neglected. In our epidemiological investigation of *E. bieneusi* in diarrheal patients, three (1.4%) of them were found to be infected with *E. bieneusi*, with one in each of the three age groups (<5 years, 5–17 years and >60 years). In China, although human epidemiological studies of *E. bieneusi* have been carried out in several populations (0.2%–22.5%), only two studies explored the relationship between *E. bieneusi* infection and ages. Children were observed to have higher prevalences of *E. bieneusi* than adults (Chongqing: 14.1% versus 7.4%; Shandong: 18.5% versus 0; Shanghai: 13.6% versus 13.3%) [24, 53]. However, similar to our study, there was no statistical difference in prevalence between children and adults. In contrast, in an investigation of *E. bieneusi* in Queensland, Australia, a statistical difference in prevalence was observed between children (4.3%, 5/115) and adults (0.6%, 1/181) [58]. Some previous studies ever recorded the high *E. bieneusi* prevalence in children, such as 24.6% in aged 13–24 months in Thailand [33] and 32.9% in aged <60 months in Uganda [43]. Like other infectious diseases, children are usually identified as a population group at risk for *E. bieneusi* infection [27]. The age-related distribution of *E. bieneusi* might be attributable to the fact that children have an immature immune system and poor hygiene habits, as well as high exposure to microsporidian infection. In the present study, all the individuals of *E. bieneusi* infection had no experience of direct contact with animals. However, some previous studies considered that people who keep livestock and live in close contact with their animals are at high risks of contracting *E. bieneusi* infections [16, 35]. In Thailand, an unusual genotype Peru16 was found in seven guinea pigs and one 2-year-old child in the same household, suggesting the possibility of zoonotic transmission of *E. bieneusi* infection [2]. The parasite was also identified in wild boars (7.7%), sika deer (8.2%) and dogs (3.2%). Previous epidemiological studies have confirmed the presence of *E. bieneusi* in the three mammal species in some provinces in China, with the prevalence ranges: 41.2%–42.0% in wild boars [10, 20], 2.2%–44.1% in sika deer [13, 42, 56, 61], and 6.0%–22.9% in dogs [3]. However, prevalences are complicated and difficult to compare. Actually, many factors are considered to possibly have influence on prevalences of *E. bieneusi*, including the health status and age of hosts, the size and structure of samples, geographical locations, farming practices and living conditions. In the present study, *E. bieneusi* was found in ostriches (10.7%) for the first time in China.

Sequence analysis of the ITS region of the rRNA gene identified nine genotypes out of 18 *E. bieneusi*-positive samples: D and YCHH1 and YCHH2 in humans (one each) and D (*n* = 4), Type IV (*n* = 1), Peru6 (*n* = 1), BEB6 (*n* = 6) and YCHA1, YCHA2 and YCHA3 (one each) in animals. Genotypes D (syn. CEbC, NCF7, Peru9, PigEBITS9, PtEb VI, SHW1, WL8) and Type IV (syn. BEB5, BEB5-var, CMITS1, K, Peru2, PtEb III, SH12) are commonly reported genotypes in human cases of *E. bieneusi* infection, and have a wide geographic distribution. To date, human infection cases with genotypes D and Type IV have been reported in at least 21 and 14 countries, respectively [21]. In China, genotypes D and Type IV have been identified in humans from six and four provinces, respectively [12, 36, 53]. The two genotypes also have an extensive host range of animals, and they have been found in numerous mammal species and bird species worldwide [21]. In China, genotypes D and Type IV have been identified in at least 22 and 7 animal species, respectively [47]. In the present study, genotype D was found in humans, one mammal species (sika deer) and one bird species (ostriches), indicating potential zoonotic transmission of human microsporidiosis caused by genotype D in the investigated areas. Although there was an absence of genotype Type IV in humans, the epidemiological role of the ostriches infected with *E. bieneusi* should be considered. In addition, we also identified genotypes Peru6 and BEB6 in sika deer, which are mainly seen in animals. Genotype Peru6 has been detected in eight mammal species and nine bird species, with six mammal species and four bird species in China [12]. Currently, together with genotypes D, EbpC, Type IV, Peru8, and Peru11, genotype Peru6 has been one of the common genotypes detected in humans [23]. In China, this genotype was reported in ethnic minority groups (Yao people) [12]. Genotype BEB6 (syn. SH5) was originally detected in cattle [9]. To date, this genotype has been identified in at least 14 mammal species and two bird species, and it is the most frequent contributor to *E. bieneusi* infections in cattle, deer, sheep, and goats [21, 23]. Genotype BEB6 has also been found in a Chinese child [45], raising public health concern related to the zoonotic potential together with other group 2 genotypes BEB4, I, and J identified in humans.

In a phylogenetical analysis, five novel genotypes of *E. bieneusi* were divided into three different genetic groups. Human-derived genotypes YCHH1 and YCHH2 and wild boar-derived genotype YCHA1 belong to group 1. Group 1 has relatively loose host specificity and the vast majority of zoonotic genotypes are in this group. Meanwhile, although some genotypes are currently found only in animals, they show a close genetic relationship to human-pathogenic genotypes, establishing the zoonotic potential of the genotypes in group 1 [21]. In the present study, genotypes YCHH1 and YCHH2 and YCHA1 were observed to have one, two and one base changes compared to zoonotic genotypes D, EbpA and D, respectively, indicating the potential for zoonotic or cross-species transmission. Dog-derived genotype YCHA2 falling into group 2 only had one base substitution compared to zoonotic genotype BEB6. Group 2 was previously composed of ruminant-specific genotypes [22]. With increasing genotyping data of *E. bieneusi*, there is a rapid increase in the number of group 2 genotypes and an extension of their host range [21]. It is worth noting that some of them (such as BEB4, BEB6, I and J) have been identified in humans, increasing their importance and concern for public health. Dog-derived genotype YHHA3 fell into group 11 and differed from genotype PtEb IX in this group by three bases.

To date, there have been 21 genotypes in group 11, and these genotypes show strong host specificity, being rarely detected in non-canine animals. Sixteen genotypes have been detected in dogs, with 13 only identified in dogs (Table 3). Currently, group 11 is thought to be the dog-specific group. A recent study revealed that genotypes PtEb IX and WW8 of group 11 are genetically far from other genotype groups at the four genetic loci (ITS, 16S rRNA, *ck1* and *swp1*) [32]. In fact, in phylogenetic analysis of ITS sequences, sequences of group 11 genotypes are often used as an outgroup. Early in 2018, genotypes MWC_m3 and MWC_m4 of group 11 were found to differ from *E. bieneusi* at the 16S rRNA gene locus, and they were suggested to belong to a new *Enterocytozoon* sp. [57].

**Table 3:** Host ranges of *E. bieneusi* genotypes of group 11.

Host	Genotypes (syn)	Country	Refs.
Dogs	CD7, CD8, WW8 (CD9), PtEb IX (eb52), NED3, NED4, GD1-GD6, SCD-1, DgEb I, DgEb II, YCHA3	China	[14, 19, 44, 50, 62], this study
	PtEb IX (eb52)	Poland; USA; Colombia; Portugal; Switzerland; Japan; Australia; Spain	[1, 5, 11, 26, 28, 34, 40, 59]
Cats	PtEb IX (eb52), GD1, GD2, WW8 (CD9), GC1	China	[44, 62]
	PtEb IX (eb52)	Poland; Australia	[34, 59]
Swans	WW8 (CD9), PtEb IX (eb52)	China	[49]
Badgers	PtEb IX (eb52)	Spain	[41]
Marsupials	MWC_m2, MWC_m3, MWC_m4	Australia	[57]
	CSK2	China	[63]

## Conclusion

The present study demonstrated the occurrence of *E. bieneusi* in patients with diarrhea and four animal species in Yichun City, with the first finding of *E. bieneusi* in ostriches in China. The finding of genotype D in humans and animals, and the identification of zoonotic genotypes Peru6, Type IV, BEB6 in animal-derived *E. bieneusi* isolates indicate the potential of zoonotic transmission of microsporidiosis caused by *E. bieneusi*. The observation of the four novel genotypes belonging to groups 1, 2 indicates a large zoonotic possibility of these genotypes and public health significance. However, in our study, there are also some limitations. The total number of fecal samples used was not large enough and the number of positive specimens was low, particularly in humans. Thus, considering the principle of “One world One health”, future molecular epidemiological surveys of *E. bieneusi* need to be carried out in different populations as well as various animal hosts and environmental samples to confirm the present conclusion of potential zoonotic transmission. A multilocus sequence typing (MLST) tool reflecting more genetic information will be used in epidemiological investigations of *E. bieneusi* transmission, especially those of public health significance of animal-derived *E. bieneusi* isolates [11]. Moreover, due to the absence of the immune status of the patients, the relationship between the immune status of the patients and *E. bieneusi* infection as well as occurrence of diarrhea will also be explored in the future by analyzing data on CD4^+^ T-lymphocyte counts of patients.
