# Supplementary material for: Enterocytozoon bieneusi in patients with diarrhea and in animals in the northeastern Chinese city of Yichun: genotyping and assessment of potential zoonotic transmission
Source: Parasite. 2022 Sep 1;29:40. doi: 10.1051/parasite/2022041 (PMC9435466; doi:10.1051/parasite/2022041)
Supplement: Supplementary file 1 — Supplementary Table S1: Distribution of E. bieneusi genotypes in humans in China. [file parasite-29-40-s1.pdf]

Table S1. Distribution of *E. bieneusi* genotypes in humans in China.

Genetic group	ITS genotypes (n)	Location	Reference
Group 1	PigEBITS5 (3), CC2 (2), **CQ-H2 (2)**, **CQ-H3 (2)**, **CQ-H1 (1)**, **CQ-H4 (1)**	Chongqing	[8]
	D (11), type IV (8), PigEBITS7 (7), EbpC (4), **GX25 (1)**, **GX456 (1)**, **GX458 (1)**	Guangxi	[24]
	EbpC (39), D (12), type IV (7), PigEBITS7 (1), Peru8 (1), EbpD (1), LW1 (1) [a], **Henan-II (1)**, Henan-III-Henan-V (1 each), Peru11 (1)	Henan	[46]
	EbpC (11), Henan-IV (3), CS-4 (2), **NEC1–NEC5 (1 each)**	Heilongjiang	[51]
	D (4), **HLJ-CP1 (1)**	Heilongjiang	[54]
	CHN4 (3)	Jilin	[55]
	Peru11 (6), SH2 (3), EbpA (2), D (1), EbpC (1), LW1 (1) [a], **SH1 (1)**, **SH3 (1)**, **SH4 (1)**, **SH6 (1)**, SH8 (1), **SH9–SH11 (1 each)**, type IV (1) [a]	Shanghai	[45]
	D (1)	Hubei	[48]
	NIA1 (19), D (6), **A (3)**, EbpA (3), **CXJH1 (1)**, KB-1 (1)	Xinjiang	[36]
	Peru 6 (21), **YN104 (1)**, **YN241 (1)**, **YN249 (1)**	Yunnan	[12]
	D (1), **CQH5 (1)**, **CQH9 (1)**, **CQH10 (1)**, **CQH11 (1)**	Chongqing, Hubei, Shandong [b]	[53]
	D (4), Macaque3 (2)[a], **MEB5 (1)**	Guangxi	[17]
	D (17), PigEBITS7 (2), CM8 (1)	Henan	[52]
Subtotal	D (57), EbpC (55), Peru6 (21), NIA1 (19), type IV (16), PigEBITS7 (10), Peru11 (7), EbpA (5), Henan-IV (4), A (3), CHN4 (3), SH2 (3), PigEBITS5 (3), CC2 (2), Macaque3 (2), **CQ-H2 (2)**, **CQ-H3 (2)**, CS-4 (2), LW1 (2), CM8 (1), **CQ-H1 (1)**, **CQ-H4 (1)**, **CQH5 (1)**, **CQH9 (1)**, **CQH10 (1)**, **CQH11 (1)**, **CXJH1 (1)**, EbpD (1), **GX25 (1)**, **GX456 (1)**, **GX458 (1)**, **Henan-II (1)**, Henan-III (1), Henan-V (1), **HLJ-CP1 (1)**, **MEB5 (1),** KB-1 (1), **NEC1–NEC5 (1 each)**, Peru8 (1), **SH1 (1)**, **SH3 (1)**, **SH4 (1)**, **SH6 (1)**, SH8 (1), **SH9–SH11 (1 each)**, **YN104 (1)**, **YN241 (1)**, **YN249 (1)**		
Group 2	BEB4 (5) [a], CHN3 (4), I (3), J (3), **CHN2 (2)**	Jilin	[55]
	BEB6 (1) [a]	Shanghai	[45]
	**CXJH 2 (1)**, **CXJH 3 (1)**, CHN6 (1)	Xinjiang	[36]
	J (2), BEB6 (1),	Henan	[52]
Subtotal	BEB4 (5), J (5), CHN3 (4), I (3), BEB6 (2), **CHN2 (2)**, CHN6 (1), **CXJH2 (1)**, **CXJH3 (1)**		
Group 5	**CQH6 (1)**, **CQH7 (1)**, **CQH8(1)**	Chongqing, Hubei, Shandong [b]	[53]

The bolded genotypes are found only in humans.

[a] Four genotypes in Table S1 have been changed to the first published names instead of genotype names described in original papers (SH5 to

BEB6, CHN1 to BEB4, SH12 to type IV, SH7 to LW1, Henan I to LW1, CM1 to Macaque3).

[b] Unspecific geographical distribution of the genotypes in one study conducted in Chongqing, Hubei, Shandong by Zang et al. (2021) [44].
